# RoFDT: Identification of Drug–Target Interactions from Protein Sequence and Drug Molecular Structure Using Rotation Forest

**DOI:** 10.3390/biology11050741

**Published:** 2022-05-13

**Authors:** Ying Wang, Lei Wang, Leon Wong, Bowei Zhao, Xiaorui Su, Yang Li, Zhuhong You

**Affiliations:** 1 College of Information Science and Engineering, Zaozhuang University, Zaozhuang 277160, China; zzxywy@uzz.edu.cn; 2 Big Data and Intelligent Computing Research Center, Guangxi Academy of Sciences, Nanning 530007, China; lghuang@gxas.cn; 3 Xinjiang Technical Institutes of Physics and Chemistry, Chinese Academy of Sciences, Urumqi 830011, China; zhaobowei19@mails.ucas.ac.cn (B.Z.); suxiaorui19@mails.ucas.ac.cn (X.S.); 4 School of Computer Science and Information Engineering, Hefei University of Technology, Hefei 230601, China; 2021010123@mail.hfut.edu.cn; 5 School of Computer Science, Northwestern Polytechnical University, Xi’an 710129, China

**Keywords:** drug, rotation forest, target protein, support vector machine

## Abstract

**Simple Summary:**

Determining the drug–target relationships is the key to modern drug development, and it plays a crucial role in drug side effects research and individual treatment. However, traditional drug target identification by bio-experimental methods is often difficult to develop due to limitations of precision, flux and cost. With the rapid development of bioinformatics and computational biology, the computer-assisted drug–target interaction (DTIs) prediction approach has attracted great attention by researchers as an accurate and quick mean of drug target recognition. In this study, combined with the protein sequence information and drug molecular structure information, a prediction method of DTIs based on machine learning is developed to achieve the purpose of locking targets and saving costs for new drug research.

**Abstract:**

As the basis for screening drug candidates, the identification of drug–target interactions (DTIs) plays a crucial role in the innovative drugs research. However, due to the inherent constraints of small-scale and time-consuming wet experiments, DTI recognition is usually difficult to carry out. In the present study, we developed a computational approach called RoFDT to predict DTIs by combining feature-weighted Rotation Forest (FwRF) with a protein sequence. In particular, we first encode protein sequences as numerical matrices by Position-Specific Score Matrix (PSSM), then extract their features utilize Pseudo Position-Specific Score Matrix (PsePSSM) and combine them with drug structure information-molecular fingerprints and finally feed them into the FwRF classifier and validate the performance of RoFDT on Enzyme, GPCR, Ion Channel and Nuclear Receptor datasets. In the above dataset, RoFDT achieved 91.68%, 84.72%, 88.11% and 78.33% accuracy, respectively. RoFDT shows excellent performance in comparison with support vector machine models and previous superior approaches. Furthermore, 7 of the top 10 DTIs with RoFDT estimate scores were proven by the relevant database. These results demonstrate that RoFDT can be employed to a powerful predictive approach for DTIs to provide theoretical support for innovative drug discovery.

## 1. Introduction

A critical step in innovative drug development is determining the interactions among drugs and targets, which is the forerunner of drug design [1,2]. Drugs play an important role in the human body by interacting with their targets, of which proteins are an essential target. By inhibiting or enhancing the function of the target protein, the drug achieves the goal of treating the disease. Although the advent of high-throughput sequencing methods has provided technical support for determining DTIs and extensive efforts has been made by drug developers, few new drugs are still approved by the Food and Drug Administration (FDA) for marketing each year [3,4,5,6]. The main reason is that the identification of DTIs by wet experimental approaches alone consumes a lot of time and money, and the scale of identification is small. With the development of computational biology, this situation can be greatly alleviated. Computer-aided prediction of DTIs can be executed rapidly at scale, providing reliable candidate drug targets for biological experiments and theoretical support for new drug development [2,4,7,8,9].

To date, computer-aided prediction-based models for DTIs have been devised by numerous researchers, and they can be roughly classified into two groups: the approach based on network and the approach based on machine learning [10,11,12]. The approach based on network approach typically characterizes the association among targets and drugs as a heterogeneous network, and predict DTI by evaluating network topology node similarity. For instance, the SDTBNI model designed by Wu et al. [13] predicts DTIs by DTI networks, drug and entity substructure linkages in unknown network space. Chu et al. [14] proposed a new DTI prediction method called the DTI-CDF model. This method can not only extract similarity features between drugs, but also extract similarity features between target proteins from heterogeneity graph, which greatly improves the prediction performance. Zhang et al. [15] designed the prediction method of DTIs according to LPLNI, which makes use of the data of neighborhood re-construction. Chu et al. [16] facilitate multi-label classification by introducing the community detection method DTI-MLCD for DTI prediction. The method performs significantly better than other machine learning methods and other existing methods in the updated gold standard dataset. Zong et al. [17] used DeepWalk combined with target-target and drug-drug similarities to accurately predict DTIs with the support of Linked Tripartite Network (LTN) and biomedical related data. The approach based on machine learning mainly uses computer to extract data features and combine with classifier to implement DTIs prediction [18,19]. For example, Peng et al. [20] used a semi-supervised inference way to predict DTIs by combining a PCA-based convex optimization algorithm with information about drug targets.

**Figure 1:**
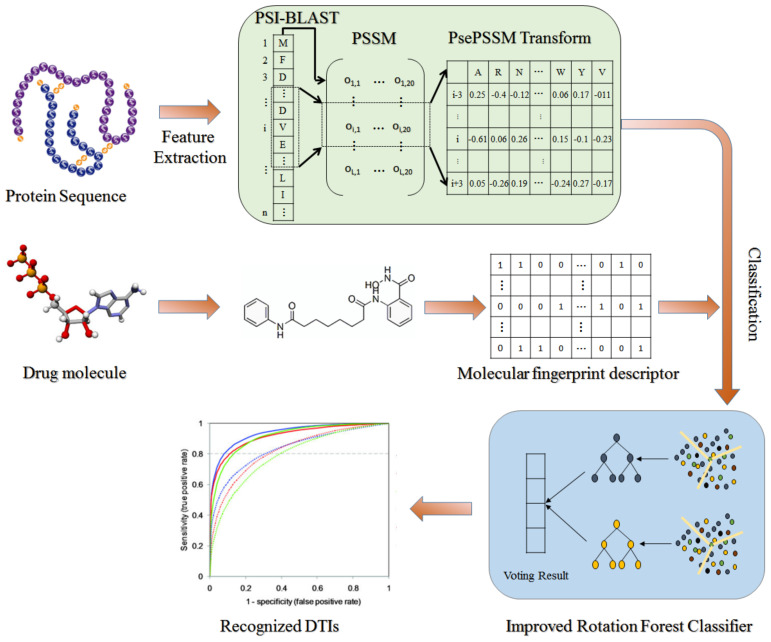
Flow framework diagram of RoFDT model.

On the basis of the hypothesis that drugs with chemical similarity have similar bioactivity, the DTIs prediction method of target protein information combined with drug structure information has achieved excellent results. Therefore, this paper designs the machine learning approach for predicting DTIs according to this hypothesis. Specifically, we first extracted the protein sequence features information using the Pseudo Position-Specific Score Matrix (PsePSSM) method, then fused them with drug molecular fingerprint descriptors and finally accurately predicted DTIs with interactions by the feature-weighted rotation forest classifier (FwRF) classifier. We tested the performance of RoFDT in datasets including Enzyme, GPCR, Ion Channel and Nuclear Receptor, and compared them with other feature approaches, classifier approaches and previous methods. The superior results demonstrate that the proposed model has excellent ability to identify DTIs. The frame diagram of rofdt is shown in Figure 1.

## 2. Materials and Methods

### 2.1. Gold Standard Datasets

In the present study, we validated the performance of RoFDT on four gold standard datasets, including Enzyme, GPCR, Ion Channel and Nuclear Receptor. These data were collected from SuperTarget & Matador [21], KEGG BRITE [22], BRENDA [23] and DrugBank [24] databases by Yamanishi et al. [25]. In these four datasets, the number of DTIs pairs (drug, target) they contain is (445, 664), (210, 204), (233, 95) and (54, 26), respectively, and the number of DTIs with interaction (positive sample) is 2926, 1476, 635 and 90, respectively [26]. We describe the network of DTIs by a bipartite diagram, where targets or drugs are presented by nodes and their associations are represented by edges. To construct the balanced dataset, we use the random strategy to select the same number of negative samples as positive samples.

### 2.2. Drug Molecular Descriptor

Drug molecular fingerprinting is widely used to characterize drug compounds because it can directly represent the association between molecular properties and structure and does not need their three-dimensional structural information. Drug molecule fingerprinting manages molecular substructures with dictionary strategy. For a particular molecule, the corresponding position of its dictionary is set to 1 when it has a certain substructure and 0 otherwise. Thus, the fingerprint descriptor of a given drug molecule can be constructed. We used molecular fingerprints from PubChem in this study, and the fingerprints property is “PUBCHEM_CACTVS_SUBGRAPHKEYS”. The compound molecule is decomposed into 881 substructures, so its fingerprint feature descriptor is also 881-dimensional.

### 2.3. Target Protein Descriptor

In this study, PSSM [27] was used to generate descriptors of protein sequences. PSSM *S(i,j)* can be characterized as $S=\left\{\partial_{i,j}:i=1\cdots L\;and\;j=1\cdots 20\right\}$, which is an L × 20 matrix, in which the length of sequence is L and the types of amino acids are 20. Therefore, the formula of *S(i,j)* is described as shown below:

(1) $$S=\left[\begin{array}{c} \begin{array}{cc} \sigma_{1,1} & \sigma_{1,2} \end{array}\;\;\;\begin{array}{cc} \cdots & \sigma_{1,20} \end{array}\\ \begin{array}{cc} \sigma_{2,1} & \sigma_{2,2} \end{array}\;\;\;\begin{array}{cc} \cdots & \sigma_{2,20} \end{array}\\ \begin{array}{cc} \vdots \;\;\;\;\;\; & \vdots \;\;\; \end{array}\;\;\;\;\begin{array}{cc} \vdots \;\;\;\;\; & \vdots \end{array}\;\;\\ \begin{array}{cc} \sigma_{L,1} & \sigma_{L,2} \end{array}\;\;\;\begin{array}{cc} \cdots & \sigma_{L,20} \end{array} \end{array}\right]$$

where $\sigma_{i,j}$ indicates the probability that the ith residue of the protein is mutated into the jth amino acid during evolution.

We obtained PSSM through Position-Specific Iterated BLAST (PSI-BLAST) according to SwansProt dataset [28,29]. PSI-BLAST will calculate the vector indicating the mutational conservatism of 20 different amino acids. To obtain broad and high homologous protein, the parameter *e*-value and iterations are set to 0.001 and 3, respectively.

### 2.4. Protein Feature Extraction

For better compatibility with the PSSM matrix, we extracted the potential features of proteins using the PsePSSM designed by Chou et al. [30], which can be denoted as below:

(2) $$e_{i,j}=\frac{e_{i,j}^{0}-\frac{1}{20}\sum_{k=1}^{20}e_{i,k}^{0}}{\sqrt{\frac{1}{20}\sum_{v=1}^{20}{\left(e_{i,v}^{0}-\frac{1}{20}\sum_{k=1}^{20}e_{i,k}^{0}\right)}^{2}}}\;i=1\ldots 20,\;j=1\ldots 20$$

where $e_{i,j}^{0}$ is the score calculated by PSI-BLAST, which score can be positive or negative. The probability of the appropriate mutation in the protein sequence higher than unexpectedly expected is indicated by a positive number, otherwise a negative number. However, since protein sequences of different lengths yield different rows of substrates, we thus need to convert them to a uniform pattern using the following equation:

(3) $${\bar{M}}_{PSSM\;=\;\left[{\bar{e}}_{1}\;{\bar{e}}_{2}\;\cdots \;{\bar{e}}_{20}\right]}$$

and:

(4) $${\bar{e}}_{j}=\frac{1}{L}\sum_{i=1}^{L}e_{i,j}\;\;\;j=1\ldots 20\;$$

where ${\bar{e}}_{j}$ represents the average score when protein residue P evolves into a *j*-type amino acid. To prevent protein P from losing its sequence information during evolution, we improved the equation by constructing pseudo-amino acids, which are described as follows:

(5) $${\bar{e}}_{j}=\{\begin{array}{c} \frac{1}{L}\overset{L}{\underset{i=1}{\sum }}e_{i,j}\;\;\;\;\;\;\;\;\;\;\;\;\;\;\;\;\;\;\;\;\;\;\;\;\;\;\;\;\;\;\;\;\;j=1\ldots 20,\;\lambda =0\;\;\;\\ \frac{1}{L\;-\;\lambda }\overset{L-\lambda }{\underset{i=1}{\sum }}{\left(e_{i,j}-e_{i+\lambda ,j}\right)}^{2}\;\;\;\;\;\;j=1\ldots 20,\;\lambda <L\;\;\; \end{array}\;$$

where $e_{j}$ is the correlation factor of *j*-type amino acids.

### 2.5. Classification Prediction

In our study, we classify and predict DTIs feature descriptors by FwRF. This classifier has the advantage of increasing the effective feature weights and removing the noise information, which can effectively improve the prediction accuracy. FwRF uses the $\chi^{2}$ statistical method to obtain the weights of different features, and its formula is as follows:

(6) $$\chi^{2}=\sum_{i=1}^{n}\sum_{j=1}^{2}\frac{{\left(\Upsilon_{ij}-\beta_{ij}\right)}^{2}}{\beta_{i,j}}$$

where $\Upsilon_{ij}$ is the number of $f_{j}$ categories with the value $v_{i}$, and its statistics are as follows:

(7) $$\Upsilon_{ij}=count\left(F=v_{i}\;and\;C=f_{j}\right)$$

$\beta_{i,j}$ is the expectation of $v_{i}$ and $f_{j}$, and it can be denoted as below:

(8) $$\beta_{i,j}=\frac{count\left(F=v_{i}\right)\times count\left(C=f_{j}\right)}{N}$$

where N is the total sample size. In feature F, the sample size whose value is $v_{i}$ is recorded as $count\left(F=v_{i}\right)$, and in class C, the sample size whose value is $f_{j}$ is recorded as $count\left(C=f_{j}\right)$.

FwRF first calculates the weights of the features using the $\chi^{2}$ statistical method, then sorts them in descending order and removes the low-weight features depending on the parameters, and finally uses the newly obtained feature set for classification prediction.

Rotation forest (RF) [31,32] is a widespread classifier. Given a dataset $\left\{x_{i},y_{i}\right\}$ containing S training samples, where $x_{i}$ is the data and $y_{i}$ is the label, the data $x_{i}$ consist of n features, thus forming a matrix of S×n. The decision tree in RF is presented as $D_{1},D_{2},\ldots ,D_{N}$, and there are N in total. The execution steps of RF are as follows.

a. The feature set F is grouped into K-independent parts of the number nk by the appropriate parameter.

b. The new matrix $X_{i,j}$ of the training set X is formed using the corresponding feature columns of $D_{i,j}$, and 3/4 of the features are selected from it forms matrix ${X^{’}}_{i,j}$ with bootstrap.

c. The coefficient matrix $M_{i,j}$ is obtained through the feature transformation ${X^{’}}_{i,j}$, and the coefficient matrix $M_{i,j}$ is rotated to generate the rotation matrix $R_{i}$, which is described as follows:

(9) $$R_{i}=\left[\begin{array}{c} \begin{array}{c} e_{i,1}^{\left(1\right)},\ldots ,e_{i,1}^{\left(N_{1}\right)}\\ 0 \end{array}\;\;\;\;\;\;\begin{array}{c} 0\;\\ e_{i,2}^{\left(1\right)},\cdots ,e_{i,2}^{\left(N_{2}\right)} \end{array}\;\;\;\;\;\;\;\;\;\;\begin{array}{c} \cdots \\ \cdots \end{array}\;\;\;\;\;\;\;\;\;\;\;\;\;\;\;\;\;\;\begin{array}{c} 0\\ 0 \end{array}\;\;\;\;\;\;\;\;\;\\ \;\;\;\;\;\;\;\;\;\;\;\;\;\begin{array}{c} \vdots \\ 0 \end{array}\;\;\;\;\;\;\;\;\;\;\;\;\;\;\;\;\;\;\;\;\;\;\;\;\;\;\;\;\begin{array}{c} \vdots \\ 0 \end{array}\;\;\;\;\;\;\;\;\;\;\;\;\;\;\;\;\;\;\;\;\;\begin{array}{c} \ddots \\ \cdots \end{array}\;\;\;\;\;\;\;\;\begin{array}{c} \vdots \;\\ e_{i,k}^{\left(1\right)},\ldots ,e_{i,k}^{\left(N_{k}\right)} \end{array} \end{array}\;\right]$$

In the classification prediction stage, the classifier $D_{i}$ calculates the confidence level $\lambda_{j}\left(x\right)$ of the test sample x using the following formula and discriminates it as the class with the highest confidence value:

(10) $$\lambda_{j}\left(x\right)=\frac{1}{N}\sum_{i=1}^{N}d_{i,j}\left(XR_{i}^{e}\right)$$

## 3. Results

### 3.1. Evaluation Indicator

To better evaluate the RoFDT performance, we used the general evaluation standard of machine learning in this study, which can be denoted as below:

(11) $$Accu.=\frac{TP+TN}{TP+TN+FP+FN}$$

(12) $$Sen.=\frac{TP}{TP+FN}\;$$

(13) $$Prec.=\frac{TP}{TP+FP}$$

(14) $$MCC=\frac{TP\times TN-FP\times FN}{\sqrt{\left(TP+FP\right)\times \left(TN+FN\right)\times \left(TP+FN\right)\times \left(TN+FP\right)}}\;$$

where *TP*, *TN*, *FP* and *FN*, respectively, represent True Positive, True Negative, False Positive and False Negative. Moreover, the receiver operating characteristic (ROC) curve [33,34,35] and area under the ROC curve (AUC) were also calculated to reflect the performance of RoFDT.

### 3.2. Parameter Evaluation

**Figure 2:**
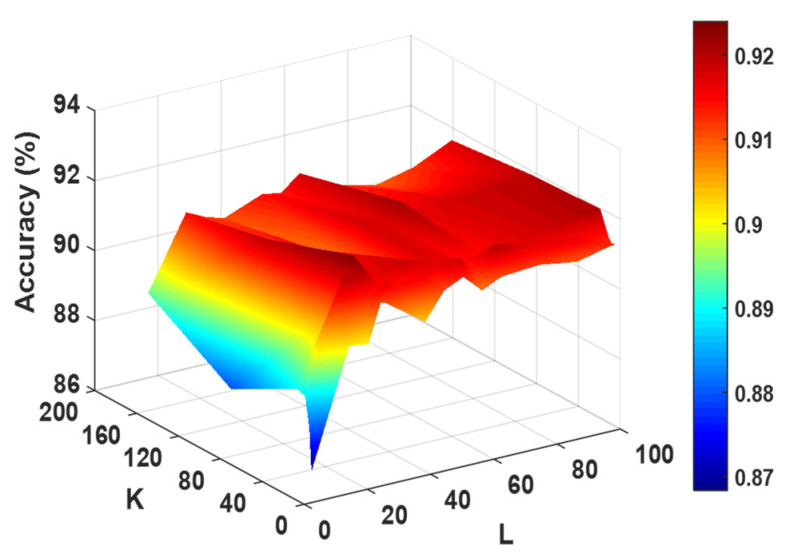
The influence of different parameters of the FwRF classifier on classification accuracy.

To maximize the RoFDT performance, the grid search approach is employed to verify the FwRF and PsePSSM parameters. When data features are extracted using the PsePSSM algorithm, the information content can be adjusted by changing the parameters in Equation (5) to obtain different feature values. We investigate the effect of different parameters of PsePSSM on the subsequent classification effect in this experiment in order to select the best combination of parameters. The effect of different parameters of the classifier on its prediction accuracy in the enzyme dataset is shown in Figure 2. It can be seen from the figure that the RF classifier achieves the highest accuracy when the feature subset K, the feature selection ratio r and the number of decision trees L are set to 16, 0.8 and 21, respectively. Therefore, we apply them as the optimal parameters in the model.

### 3.3. Prediction Performance Evaluation

**Figure 3:**
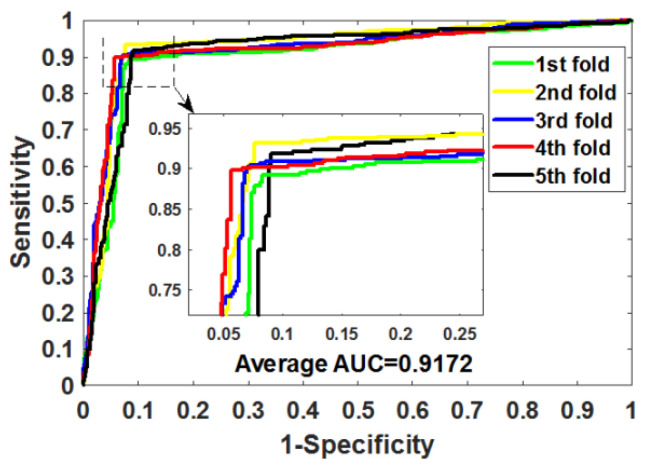
ROC curves of the 5FCV experiment acquired by RoFDT on the Enzyme dataset.

**Figure 4:**
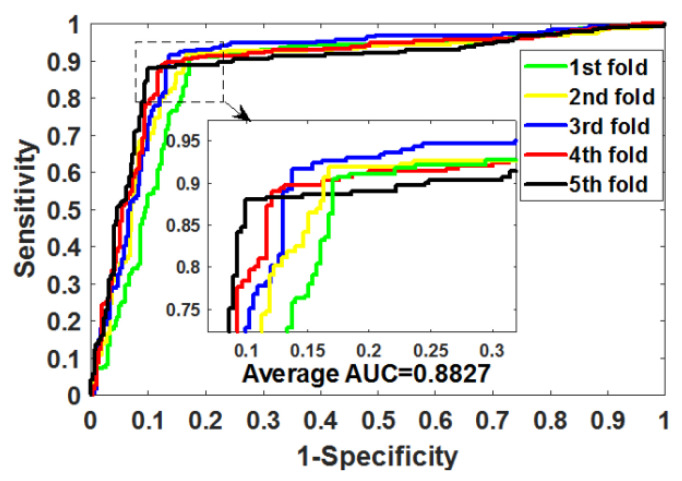
ROC curves of the 5FCV experiment acquired by RoFDT on the Ion Channel dataset.

**Figure 5:**
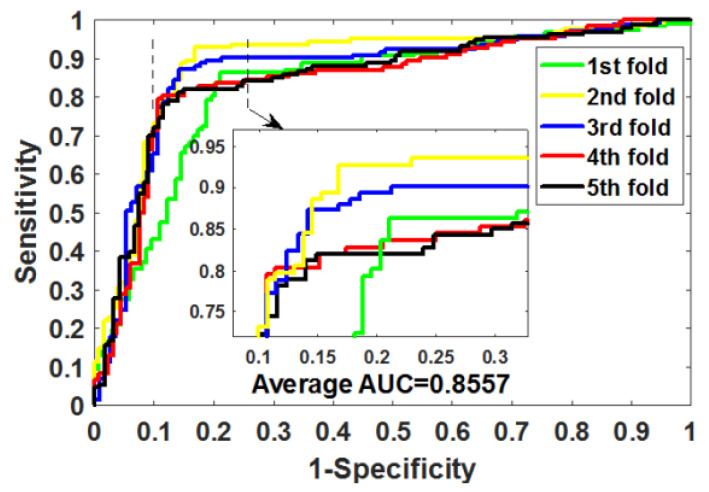
ROC curves of the 5FCV experiment acquired by RoFDT on the GPCR dataset.

**Figure 6:**
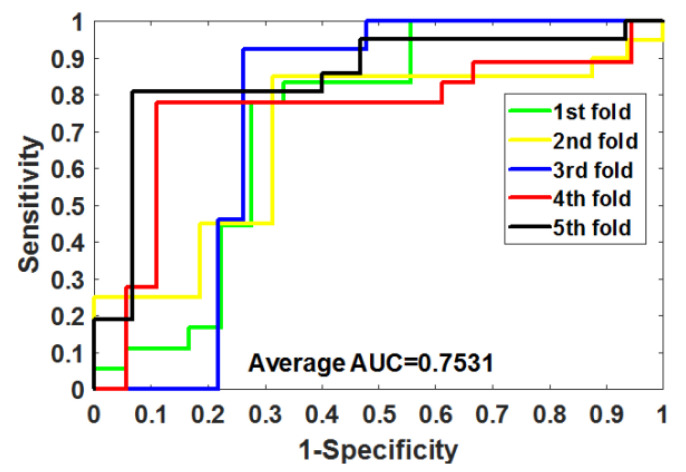
ROC curves of the 5FCV experiment acquired by RoFDT on the Nuclear Receptor dataset.

After optimizing the parameters of RoFDT, we evaluate its performance in the Enzyme, GPCR, Ion Channel and Nuclear Receptor datasets using the five-fold cross-validation (5FCV) method. Table 1, Table 2, Table 3 and Table 4 summarizes the outcomes obtained by RoFDT on the gold standard datasets. In these datasets, RoFDT achieved 91.68%, 88.11%, 84.72% and 78.33% prediction accuracy, and its standard variance was 0.84%, 1.01%, 1.94% and 5.34%, respectively. In terms of sensitivity, RoFDT achieved scores of 90.84%, 90.30%, 84.73% and 81.97% in the four datasets with standard variances of 1.68%, 1.61%, 3.45% and 7.85%, respectively. RoFDT achieved 83.39%, 79.02%, 74.06%, 65.56% and 91.72%, 88.27%, 85.57% and 75.31% in the MCC and AUC evaluation metrics, which combine to show predictive performance, respectively. These excellent results show that RoFDT has good ability to predict DTIs with strong robustness. Figure 3, Figure 4, Figure 5 and Figure 6 show the ROC curves acquired by RoFDT in the gold standard dataset, respectively.

### 3.4. Comparison of Different Feature Models

To estimate the influence of the PsePSSM algorithm on the RoFDT model, we compare it with the Local Phase Quantization (LPQ) algorithm model on four gold standard datasets in this part of the experiment. The LPQ algorithm originally described in the article for texture description by Ojansivu and Heikkila [36] and is according to the blur invariance property of the Fourier phase spectrum [37,38,39]. Table 5 lists the 5FCV outcomes produced by LPQ combined with FwRF on gold standard datasets. As observed in Table 5, RoFDT has gained the optimal outcomes in all evaluation indicators. Detailed 5FCV outcomes on four gold standard datasets are aggregated in Tables S1–S4 of Supplementary Materials. For a fair comparison, FwRF was set with the same hyperparameters in the experiment. From the experimental outcomes, it can be seen that PsePSSM combined with FwRF can effectively promote the model performance.

### 3.5. Classifier Model Comparison

To investigate further the influence of various classifiers on the RoFDT performance, we compare it with the SVM classifier model. The parameters of the SVM were refined, and its hyperparameters g and c were optimized to 0.6 and 0.5, respectively. The optimization outcomes of SVM parameters are shown in detail in Table S9 of the Supplementary Materials. As can be seen in Table 6, RoFDT achieved higher scores in all four gold standard datasets compared to the SVM model. Specifically, RoFDT achieved optimal results in the four gold standard datasets for accuracy, MCC, sensitivity and AUC, but was only slightly less precision than the SVM model in the Ion Channel and Enzyme datasets. Detailed 5FCV experimental outcomes on gold standard datasets are shown in Tables S5–S8 of Supplementary Materials. The experimental results of comparing different classifier models show that the FwRF classifier used by RoFDT can be better compatible with it, which helps to increase the model prediction accuracy.

### 3.6. Comparison with Previous Models

Using the powerful computing power of computer to predict DTIs on a large scale has become increasingly important in the field of new drug research and development. Numerous researchers have constructed different computational models to solve this problem. To further evaluating RoFDT’s capabilities, we compare it with these excellent models. Among these excellent models, we chose the model that is also implemented in the four datasets and evaluated using 5FCV. The AUCs generated by these models are listed in Table 7. As seen in table, RoFDT performed well overall, achieving the best results on Enzyme and the second highest outcomes on Ion Channel and GPCR. However, constrained by the sample size of Nuclear Receptor, RoFDT is not sufficiently trained and performs generally in it.

### 3.7. Case Studies

To verify the power of RoFDT to predict unknown DTIs, all known DTI pairs are used to train RoFDT and predict in its unknown space. We validate the top 10 DTIs with the highest prediction scores in SuperTarget [21] and the drug target pairs validated in the SuperTarget database do not contain the data used for training. SuperTarget is a drug target database with a collection of 332,828 DTIs. The outcomes of the case studies are listed in Table 8, where 7 of the top 10 with the best prediction scores were validated by this database. The case study reveals that RoFDT has the capability to competitively predict unknown DTIs. It is interesting to note that although the remaining three pairs of DTIs are not substantiated by the current database, there is a possibility that their relationship will be proved as the study progresses.

## 4. Discussion

In the present study, we propose a reliable DTI prediction approach RoFDT by combining protein sequence and drug molecular structure. We first transformed the protein sequence information numerically by PSSM based on its sequence information, and extracted its hidden features using PsePSSM. The drug structure is then encoded as the digital descriptor based on molecular fingerprinting techniques. Finally, the performance of RoFDT was verified using FwRF on four benchmark datasets, and its prediction results were confirmed by the authoritative databases. All these exceptional outcomes show that RoFDT is a valid approach for predicting DTIs and can provide new insights for potential drug discovery.

RoFDT exhibits competitive advantages over previous DTI prediction models. The reason for this is that RoFDT considers that protein sequences provide rich information support for DTI prediction, and its PSSM descriptors are well compatible with PsePSSM feature extraction method to extract its potential features to the maximum extent. In addition, the molecular fingerprint descriptors of drug structures can faithfully represent different drug substructure properties, and thus, have a high characterization capability. Under the above circumstances, RoFDT was able to predict DTI more accurately and provide a more reliable theoretical basis for drug development.

However, RoFDT still has some limitations. For example, the utilization of protein sequence information by RoFDT relies mainly on PSSM, and its richer description needs to be further explored. Second, although the feature extraction method used by RoFDT has achieved better results, it still requires more manual experience to support, and the automation process needs to be better improved. Finally, RoFDT requires more data for training and is not very sensitive to newly discovered drug targets. In future research, we intend to explore more intelligent feature characterization methods to overcome the above-mentioned shortcomings and further enhance the RoFDT performance.

## 5. Conclusions

As a pioneering step in drug development, the reliable prediction and identification of DTIs plays an essential element in innovative drug research. In the present study, we combined protein sequence and drug molecular structure to design a computational model for DTIs prediction. The proposed model achieves excellent results in the gold standard datasets including Enzyme, GPCR, Ion Channel and Nuclear Receptor. The model also exhibits strong powerful in comparison with extraction algorithm models, classifier models, and previous methods. In addition, 7 of the top 10 DTIs predicted by the proposed model have been verified by relevant database. These outcomes suggest that the RoFDT model can be employed as a stable and dependable tool to provide valuable target candidates for innovative drug research.

## Figures and Tables

**Table 1:** 5FCV prediction results obtained by RoFDT on the Enzyme dataset.

Test Set	Accu.(%)	Sen.(%)	Prec.(%)	MCC(%)	AUC(%)
1	90.51	89.20	91.27	81.03	90.04
2	92.82	93.22	92.59	85.64	92.96
3	91.62	90.19	92.74	83.28	92.09
4	91.97	89.68	94.40	84.05	91.79
5	91.47	91.90	90.96	82.94	91.73
Average	91.68 ± 0.84	90.84 ± 1.68	92.39 ± 1.37	83.39 ± 1.68	91.72 ± 1.06

**Table 2:** 5FCV prediction results obtained by RoFDT on the Ion Channel dataset.

Test Set	Accu.(%)	Sen.(%)	Prec.(%)	MCC(%)	AUC(%)
1	86.61	90.38	83.76	76.76	86.16
2	87.63	91.92	84.78	78.22	87.83
3	88.98	91.61	87.22	80.36	89.68
4	88.31	89.67	87.62	79.33	89.07
5	89.02	87.93	89.47	80.43	88.59
Average	88.11 ± 1.01	90.30 ± 1.61	86.57 ± 2.29	79.02 ± 1.55	88.27 ± 1.36

**Table 3:** 5FCV prediction results obtained by RoFDT on the GPCR dataset.

Test Set	Accu.(%)	Sen.(%)	Prec.(%)	MCC(%)	AUC(%)
1	82.28	86.21	77.52	70.73	82.86
2	87.01	88.62	85.16	77.38	88.82
3	86.22	86.52	88.41	76.00	86.72
4	84.63	80.33	86.73	73.83	84.37
5	83.46	81.95	85.83	72.37	85.11
Average	84.72 ± 1.94	84.73 ± 3.45	84.73 ± 4.21	74.06 ± 2.68	85.57 ± 2.28

**Table 4:** 5FCV prediction results obtained by RoFDT on the Nuclear Receptor dataset.

Test Set	Accu.(%)	Sen.(%)	Prec.(%)	MCC(%)	AUC(%)
1	69.44	83.33	65.22	55.90	72.22
2	77.78	85.00	77.27	64.34	69.69
3	80.56	92.31	66.67	67.47	74.25
4	83.33	77.78	87.50	72.05	75.31
5	80.56	71.43	93.75	68.03	85.08
Average	78.33 ± 5.34	81.97 ± 7.85	78.08 ± 12.56	65.56 ± 6.05	75.31 ± 5.87

**Table 5:** 5FCV outcomes of the LPQ combined with FwRF model on the four gold standard datasets.

Dataset	Model	Accu.(%)	Sen.(%)	Prec.(%)	MCC(%)	AUC(%)
Enzyme	LPQ	89.63 ± 0.39	89.69 ± 1.82	89.64 ± 2.16	79.32 ± 0.79	89.40 ± 0.98
	RoFDT	91.68 ± 0.84	90.84 ± 1.68	92.39 ± 1.37	83.39 ± 1.68	91.72 ± 1.06
Ion Channel	LPQ	83.97 ± 2.32	86.93 ± 3.03	81.89 ± 3.66	68.13 ± 4.54	84.66 ± 2.01
	RoFDT	88.11 ± 1.01	90.30 ± 1.61	86.57 ± 2.29	79.02 ± 1.55	88.27 ± 1.36
GPCR	LPQ	82.52 ± 2.17	83.87 ± 3.58	81.79 ± 3.78	65.19 ± 4.15	83.19 ± 1.79
	RoFDT	84.72 ± 1.94	84.73 ± 3.45	84.73 ± 4.21	74.06 ± 2.68	85.57 ± 2.28
Nuclear Receptor	LPQ	66.67 ± 7.35	67.64 ± 16.23	67.97 ± 9.98	35.46 ± 10.89	69.56 ± 6.85
	RoFDT	78.33 ± 5.34	81.97 ± 7.85	78.08 ± 12.56	65.56 ± 6.05	75.31 ± 5.87

**Table 6:** 5FCV outcomes of different classifier models on the four gold standard datasets.

Dataset	Model	Accu.(%)	Sen.(%)	Prec.(%)	MCC(%)	AUC(%)
Enzyme	SVM	84.20 ± 0.60	69.90 ± 1.70	98.00 ± 0.50	71.50 ± 1.00	84.30 ± 1.20
	RoFDT	91.68 ± 0.84	90.84 ± 1.68	92.39 ± 1.37	83.39 ± 1.68	91.72 ± 1.06
Ion Channel	SVM	81.90 ± 1.20	69.70 ± 3.70	92.40 ± 2.20	66.00 ± 1.90	81.70 ± 1.20
	RoFDT	88.11 ± 1.01	90.30 ± 1.61	86.57 ± 2.29	79.02 ± 1.55	88.27 ± 1.36
GPCR	SVM	70.00 ± 2.10	50.40 ± 7.80	82.30 ± 3.30	42.80 ± 4.90	70.10 ± 2.70
	RoFDT	84.72 ± 1.94	84.73 ± 3.45	84.73 ± 4.21	74.06 ± 2.68	85.57 ± 2.28
Nuclear Receptor	SVM	63.30 ± 3.60	57.60 ± 7.90	67.50 ± 14.60	29.60 ± 7.40	61.80 ± 5.80
	RoFDT	78.33 ± 5.34	81.97 ± 7.85	78.08 ± 12.56	65.56 ± 6.05	75.31 ± 5.87

**Table 7:** Comparison with previous excellent models on the four gold standard dataset.

Dataset	NetCBP [40]	KBMF2K [41]	RFDT [26]	SIMCOMP [42]	RoFDT
Enzyme	0.8251	0.832	0.915	0.863	0.9172
Ion Channel	0.8034	0.799	0.890	0.776	0.8827
GPCR	0.8235	0.857	0.845	0.867	0.8557
Nuclear Receptor	0.8394	0.824	0.723	0.856	0.7531

**Table 8:** Top 10 DTI pairs predicted by RoFDT on the SuperTarget database.

Drug Name	Drug ID	Target Protein Name	Target Protein ID	Validation Database
Dihydroxypropyltheophylline	D00691	PDE7A_HUMAN	has5150	SuperTarget
Isotretinoino	D00348	RXRA_HUM	hsa6256	SuperTarget
Xanthotoxine	D00139	CP1A1_hasAN	hsa1543	SuperTarget
Loxapinsuccinate	D02340	DRhasHUMAN	hsa1812	SuperTarget
Prochlorpermazine	D00493	has2A_HUMAN	hsa3356	unconfirmed
Bromochlorotrifluoroethane	D00542	CP2E1_HUMAN	hsa1571	SuperTarget
Mifepristone	D00585	ESR1_HUMAN	hsa2099	SuperTarget
Olanzapine	D00454	DRD2_HUMAN	hsa1813	unconfirmed
Transdermal Nicotine	D03365	ACHA4_HUMAN	hsa1137	SuperTarget
Epoprostenol	D00106	PE2R3_HUMAN	hsa5733	unconfirmed

## Data Availability

Not applicable.

## Supplementary Materials

The following supporting information can be downloaded at: https://www.mdpi.com/article/10.3390/biology11050741/s1, Table S1: 5FCV results of FwRF combined with LPQ on Enzyme dataset, Table S2: 5FCV results of FwRF combined with LPQ on Ion Channel dataset, Table S3: 5FCV results of FwRF combined with LPQ on GPCR dataset, Table S4: 5FCV results of FwRF combined with LPQ on Nuclear Receptor dataset, Table S5: 5FCV results of the SVM classifier model on enzyme dataset, Table S6: 5FCV results of SVM classifier model on Ion Channel dataset, Table S7: 5FCV results of SVM classifier model on GPCR dataset, Table S8: 5FCV results of SVM classifier model on Nuclear Receptor dataset, Table S9: The results of SVM parameter optimization using grid search method on Enzyme dataset, Figure S1: The effect of different PsePSSM Parameters on classifier performance, Figure S2: The effect of different feature selection ratio on classifier performance.

## References

[1] Xia Z., Wu L.-Y., Zhou X., Wong S.T.C. (2010). Semi-supervised drug-protein interaction prediction from heterogeneous biological spaces. BMC Syst. Biol..

[2] Wang Y.-C., Yang Z.-X., Wang Y., Deng N.-Y. (2010). Computationally Probing Drug-Protein Interactions via Support Vector Machine. Lett. Drug Des. Discov..

[3] Landry Y., Gies J.-P. (2008). Drugs and their molecular targets: An updated overview. Fundam. Clin. Pharmacol..

[4] Li Q., Lai L. (2007). Prediction of potential drug targets based on simple sequence properties. BMC Bioinform..

[5] Van de Waterbeemd H., Gifford E. (2003). ADMET in silico modelling: Towards prediction paradise?. Nat. Rev. Drug Discov..

[6] Wang L., You Z.-H., Huang D.-S., Li J.-Q. (2021). MGRCDA: Metagraph Recommendation Method for Predicting CircRNA-Disease Association. IEEE Trans. Cybern..

[7] Kuruvilla F.G., Shamji A.F., Sternson S.M., Hergenrother P.J., Schreiber S.L. (2002). Dissecting glucose signalling with diversity-oriented synthesis and small-molecule microarrays. Nature.

[8] Haggarty S.J., Koeller K.M., Wong J.C., Butcher R.A., Schreiber S.L. (2003). Multidimensional chemical genetic analysis of diversity-oriented synthesis-derived deacetylase inhibitors using cell-based assays. Chem. Biol..

[9] Wang L., Yan X., You Z.-H., Zhou X., Li H.-Y., Huang Y.-A. (2021). SGANRDA: Semi-supervised generative adversarial networks for predicting circRNA–disease associations. Brief. Bioinform..

[10] Chen X., Yan C.C., Zhang X., Zhang X., Dai F., Yin J., Zhang Y. (2016). Drug-target interaction prediction: Databases, web servers and computational models. Brief. Bioinform..

[11] Alguwaizani S., Park B., Zhou X., Huang D.-S., Han K. (2018). Predicting interactions between virus and host proteins using repeat patterns and composition of amino acids. J. Healthc. Eng..

[12] Wang L., You Z.-H., Li J.-Q., Huang Y.-A. (2020). IMS-CDA: Prediction of CircRNA-Disease Associations From the Integration of Multisource Similarity Information With Deep Stacked Autoencoder Model. IEEE Trans. Cybern..

[13] Wu Z., Cheng F., Li J., Li W., Liu G., Tang Y. (2017). SDTNBI: An integrated network and chemoinformatics tool for systematic prediction of drug–target interactions and drug repositioning. Brief. Bioinform..

[14] Chu Y., Kaushik A.C., Wang X., Wang W., Zhang Y., Shan X., Salahub D.R., Xiong Y., Wei D.-Q. (2021). DTI-CDF: A cascade deep forest model towards the prediction of drug-target interactions based on hybrid features. Brief. Bioinform..

[15] Zhang W., Chen Y., Li D. (2017). Drug-Target Interaction Prediction through Label Propagation with Linear Neighborhood Information. Molecules.

[16] Chu Y., Shan X., Chen T., Jiang M., Wang Y., Wang Q., Salahub D.R., Xiong Y., Wei D.-Q. (2021). DTI-MLCD: Predicting drug-target interactions using multi-label learning with community detection method. Brief. Bioinform..

[17] Zong N., Kim H., Ngo V., Harismendy O. (2017). Deep Mining Heterogeneous Networks of Biomedical Linked Data to Predict Novel Drug-Target Associations. Bioinformatics.

[18] Huang D.-S., Zhang L., Han K., Deng S., Yang K., Zhang H. (2014). Prediction of protein-protein interactions based on protein-protein correlation using least squares regression. Curr. Protein Pept. Sci..

[19] Xia J.-F., Han K., Huang D.-S. (2010). Sequence-Based Prediction of Protein-Protein Interactions by Means of Rotation Forest and Autocorrelation Descriptor. Protein Pept. Lett..

[20] Peng L., Liao B., Zhu W., Li Z., Li K. (2017). Predicting Drug-Target Interactions With Multi-Information Fusion. IEEE J. Biomed. Health Inform..

[21] Gunther S., Kuhn M., Dunkel M., Campillos M., Senger C., Petsalaki E., Ahmed J., Urdiales E.G., Gewiess A., Jensen L.J. (2008). SuperTarget and Matador: Resources for exploring drug-target relationships. Nucleic Acids Res..

[22] Kanehisa M., Goto S., Hattori M., Aoki-Kinoshita K.F., Itoh M., Kawashima S., Katayama T., Araki M., Hirakawa M. (2006). From genomics to chemical genomics: New developments in KEGG. Nucleic Acids Res..

[23] Schomburg I., Chang A., Ebeling C., Gremse M., Heldt C., Huhn G., Schomburg D. (2004). BRENDA, the enzyme database: Updates and major new developments. Nucleic Acids Res..

[24] Wishart D.S., Knox C., Guo A.C., Cheng D., Shrivastava S., Tzur D., Gautam B., Hassanali M. (2008). DrugBank: A knowledgebase for drugs, drug actions and drug targets. Nucleic Acids Res..

[25] Yamanishi Y., Araki M., Gutteridge A., Honda W., Kanehisa M. (2008). Prediction of drug-target interaction networks from the integration of chemical and genomic spaces. Bioinformatics.

[26] Wang L., You Z.H., Chen X., Yan X., Liu G., Zhang W. (2018). RFDT: A Rotation Forest-based Predictor for Predicting Drug-Target Interactions Using Drug Structure and Protein Sequence Information. Curr. Protein Pept. Sci..

[27] Gribskov M., McLachlan A.D., Eisenberg D. (1987). Profile analysis: Detection of distantly related proteins. Proc. Natl. Acad. Sci. USA.

[28] Altschul S.F., Madden T.L., Schaffer A.A., Zhang J., Zhang Z., Miller W., Lipman D.J. (1997). Gapped BLAST and PSI-BLAST: A new generation of protein database search programs. Nucleic Acids Res..

[29] Wang L., You Z.-H., Xia S.-X., Liu F., Chen X., Yan X., Zhou Y. (2017). Advancing the prediction accuracy of protein-protein interactions by utilizing evolutionary information from position-specific scoring matrix and ensemble classifier. J. Theor. Biol..

[30] Chou K.C. (2001). Prediction of protein cellular attributes using pseudo-amino acid composition. Proteins Struct. Funct. Genet..

[31] Rodriguez J.J., Kuncheva L.I. (2006). Rotation forest: A new classifier ensemble method. IEEE Trans. Pattern Anal. Mach. Intell..

[32] Wang L., You Z.-H., Yan X., Xia S.-X., Liu F., Li L.-P., Zhang W., Zhou Y. (2018). Using Two-dimensional Principal Component Analysis and Rotation Forest for Prediction of Protein-Protein Interactions. Sci. Rep..

[33] Zweig M.H., Campbell G. (1993). Receiver-operating characteristic (ROC) plots: A fundamental evaluation tool in clinical medicine. Clin. Chem..

[34] Wang L., Wang H.-F., Liu S.-R., Yan X., Song K.-J. (2019). Predicting Protein-Protein Interactions from Matrix-Based Protein Sequence Using Convolution Neural Network and Feature-Selective Rotation Forest. Sci. Rep..

[35] Wang B., Huang D.-S., Jiang C. (2014). A new strategy for protein interface identification using manifold learning method. IEEE Trans. Nanobiosci..

[36] Ojansivu V., Heikkila J. (2008). Blur insensitive texture classification using local phase quantization. Image Signal Process..

[37] Wang H., Song A., Li B., Xu B., Li Y. (2015). Psychophysiological classification and experiment study for spontaneous EEG based on two novel mental tasks. Technol. Health Care.

[38] Li Y., Olson E.B. (2010). A General Purpose Feature Extractor for Light Detection and Ranging Data. Sensors.

[39] Li Y., Olson E.B. Structure Tensors for General Purpose LIDAR Feature Extraction. Proceedings of the 2011 IEEE International Conference on Robotics and Automation (ICRA).

[40] Chen H., Zhang Z. (2013). A Semi-Supervised Method for Drug-Target Interaction Prediction with Consistency in Networks. PLoS ONE.

[41] Gonen M. (2012). Predicting drug-target interactions from chemical and genomic kernels using Bayesian matrix factorization. Bioinformatics.

[42] Öztürk H., Ozkirimli E., Özgür A. (2016). A comparative study of SMILES-based compound similarity functions for drug-target interaction prediction. BMC Bioinform..

