# Supplementary material for: RoFDT: Identification of Drug–Target Interactions from Protein Sequence and Drug Molecular Structure Using Rotation Forest
Source: Biology (Basel). 2022 May 13;11(5):741. doi: 10.3390/biology11050741 (PMC9138819; doi:10.3390/biology11050741)
Supplement: Supplementary file 1 [file biology-11-00741-s001.zip › biology-1706366-supplementary.pdf]

Table S1: 5FCV results of FwRF combined with LPQ on *Enzyme* data set

Test set	Accu.(%)	Sen.(%)	Prec.(%)	MCC(%)	AUC(%)
1	89.91	87.64	92.52	79.96	88.94
2	89.40	89.17	90.07	78.80	88.61
3	90.17	91.23	88.54	80.36	91.09
4	89.40	88.49	90.33	78.82	89.30
5	89.25	91.92	86.73	78.65	89.08
Average	**89.63±0.39**	**89.69±1.82**	**89.64±2.16**	**79.32±0.79**	**89.40±0.98**

Table S2: 5FCV results of FwRF combined with LPQ on *Icon Channel* data set

Test set	Accu.(%)	Sen.(%)	Prec.(%)	MCC(%)	AUC(%)
1	86.61	87.95	86.54	73.17	87.79
2	84.41	87.50	82.48	68.94	84.03
3	85.42	88.70	83.70	70.93	85.33
4	82.68	88.89	77.04	66.19	83.56
5	80.71	81.60	79.69	61.44	82.57
Average	**83.97±2.23**	**86.93±3.03**	**81.89±3.66**	**68.13±4.54**	**84.66±2.01**

Table S3: 5FCV results of FwRF combined with LPQ on *GPCR* data set

Test set	Accu.(%)	Sen.(%)	Prec.(%)	MCC(%)	AUC(%)
1	80.71	78.63	83.06	61.54	80.64
2	81.10	87.80	76.60	62.97	83.34
3	84.25	84.17	86.67	68.35	83.04
4	85.43	86.32	82.79	70.83	85.69
5	81.10	82.40	79.84	62.24	83.24
Average	**82.52±2.17**	**83.87±3.58**	**81.79±3.78**	**65.19±4.15**	**83.19±1.79**

Table S4: 5FCV results of FwRF combined with LPQ on *Nuclear Receptor* data set

Test set	Accu.(%)	Sen.(%)	Prec.(%)	MCC(%)	AUC(%)
1	63.89	85.71	52.17	36.25	74.68
2	63.89	61.90	72.22	28.17	70.16
3	77.78	75.00	64.29	52.38	76.04
4	69.44	72.73	76.19	36.60	68.18
5	58.33	42.86	75.00	23.90	58.73
Average	**66.67±7.35**	**67.64±16.23**	**67.97±9.98**	**35.46±10.89**	**69.56±6.85**

Table S5: 5FCV results of the SVM classifier model on *Enzyme* data set

Test set	Accu.(%)	Sen.(%)	Prec.(%)	MCC(%)	AUC(%)
1	83.59	67.60	98.48	70.44	83.36
2	84.27	70.51	97.65	71.47	83.70
3	85.04	71.77	97.43	72.57	85.69
4	84.53	70.88	98.61	72.32	85.46
5	83.70	68.62	97.79	70.46	83.10
Average	**84.20±0.60**	**69.90±1.70**	**98.00±0.50**	**71.50±1.00**	84.30**±1.20**

Table S6: 5FCV results of SVM classifier model on *Icon Channel* data set

Test set	Accu.(%)	Sen.(%)	Prec.(%)	MCC(%)	AUC(%)
1	82.54	72.51	90.17	66.24	80.98
2	81.86	71.38	90.60	65.28	80.80
3	79.83	64.09	94.09	63.13	80.84

4	82.88	72.67	91.98	67.42	83.54
5	82.60	67.93	95.17	67.74	82.52
Average	**81.90±1.20**	**69.70±3.70**	**92.40±2.20**	**66.00±1.90**	**81.70±1.20**

Table S7: 5FCV results of SVM classifier model on *GPCR* data set

Test set	Accu.(%)	Sen.(%)	Prec.(%)	MCC(%)	AUC(%)
1	66.93	38.79	77.59	34.86	66.00
2	72.05	51.22	85.14	47.10	71.00
3	70.87	60.28	82.52	44.89	73.30
4	71.26	48.36	85.51	45.81	69.52
5	68.90	53.38	80.68	41.28	70.84
Average	**70.00±2.10**	**50.40±7.80**	**82.30±3.30**	**42.80±4.90**	**70.10±2.70**

Table S8: 5FCV results of SVM classifier model on *Nuclear Receptor* data set

Test set	Accu.(%)	Sen.(%)	Prec.(%)	MCC(%)	AUC(%)
1	61.11	55.56	62.50	22.36	66.67
2	66.67	60.00	75.00	35.00	57.81
3	58.33	69.23	45.00	20.69	55.18
4	66.67	55.56	71.43	34.19	60.49
5	63.89	47.62	83.33	35.86	68.89
Average	**63.30±3.60**	**57.60±7.90**	**67.50±14.60**	**29.60±7.40**	**61.80±5.80**

Table S9: The results of SVM parameter optimization using grid search method on *Enzyme* data set

c \ g	0.1	0.2	0.3	0.4	0.5	0.6	0.7	0.8	0.9
0.1	0.8022	0.8131	0.7103	0.6470	0.6231	0.6051	0.5915	0.5735	0.5590
0.2	0.8093	0.8135	0.8167	0.8341	0.8381	0.8316	0.7795	0.6932	0.6479
0.3	0.8124	0.8169	0.8218	0.8350	0.8373	0.8436	0.8368	0.8274	0.8222

0.4	0.8132	0.8169	0.8218	0.8375	0.8381	0.8444	0.8376	0.8274	0.8231
0.5	0.8170	0.8195	0.8235	0.8334	0.8373	0.8453	0.8402	0.8299	0.8239
0.6	0.8152	0.8195	0.8278	0.8334	0.8373	0.8452	0.8393	0.8299	0.8239
0.7	0.8159	0.8129	0.8295	0.8309	0.8390	0.8450	0.8410	0.8299	0.8248
0.8	0.8155	0.8118	0.8238	0.8292	0.8381	0.8447	0.8419	0.8333	0.8256
0.9	0.8106	0.8113	0.8221	0.8292	0.8373	0.8438	0.8410	0.8333	0.8274

Model parameters optimization

The experimental results are illustrated in Figure S1. From Figure S1, we obtained optimal parameters of λ = 34. The FwRF classifier needs to set the feature selection ratio r. Due to the feature vector is composed by molecular structure fingerprints and protein information, in order to prevent lost one of them， we set the range of feature selection ratios from 0.6 to 1. Figure S2 depicts the effect of feature selection rate on classifier performance. According to Figure S2, which illustrates the relationship between the performance of the classifier and feature selection ratio, the optimal choice is 80% in this experiment.

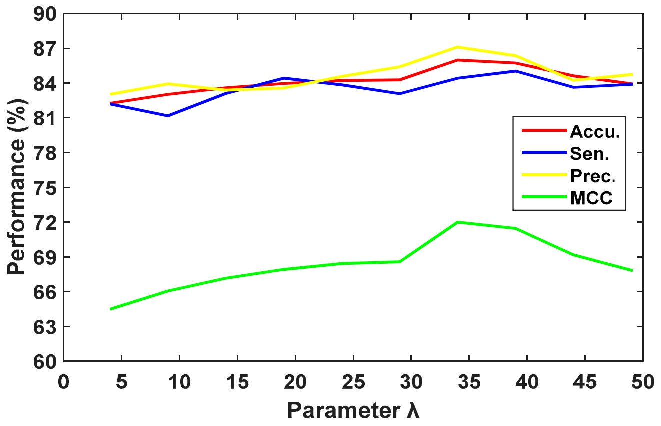

Figure S1. The effect of different PsePSSM Parameters on classifier performance

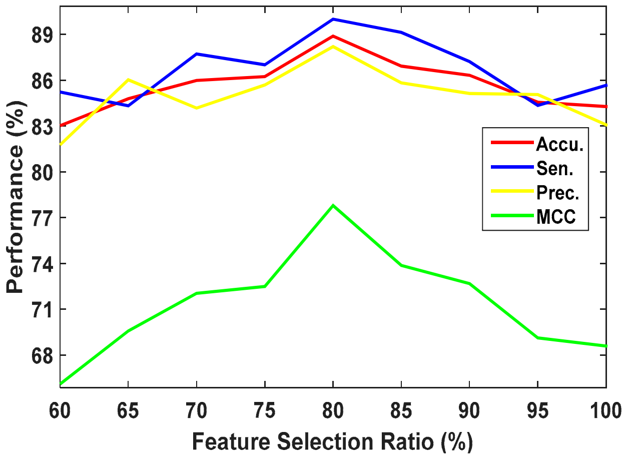

Figure S2. The effect of different feature selection ratio on classifier performance
